# An analysis of the utilisation of chemoprophylaxis against *Pneumocystis jirovecii* pneumonia in patients with malignancy receiving corticosteroid therapy at a cancer hospital

**DOI:** 10.1038/sj.bjc.6602412

**Published:** 2005-02-22

**Authors:** L J Worth, M J Dooley, J F Seymour, L Mileshkin, M A Slavin, K A Thursky

**Affiliations:** 1 Victorian Infectious Diseases Service, Centre for Clinical Research Excellence in Infectious Diseases, Grattan Street, Parkville, Victoria 3050, Australia; 2 Department of Haematology & Medical Oncology, Peter MacCallum Cancer Centre, St Andrews Place, East Melbourne, Victoria 3002, Australia; 3 Department of Pharmacy, Peter MacCallum Cancer Centre, St Andrews Place, East Melbourne, Victoria 3002, Australia; 4 Department of Pharmacy Practice, Victorian College of Pharmacy, Monash University, Royal Parade, Parkville, Victoria 3052, Australia

**Keywords:** *Pneumocystis* pneumonia, chemoprophylaxis, malignancy, corticosteroid, guidelines

## Abstract

*Pneumocystis jirovecii* pneumonia (PCP) is associated with high mortality in immunocompromised patients without human immunodeficiency virus infection. However, chemoprophylaxis is highly effective. In patients with solid tumours or haematologic malignancy, several risk factors for developing PCP have been identified, predominantly corticosteroid therapy. The aims of this study were to identify the potentially preventable cases of PCP in patients receiving corticosteroid therapy at a tertiary care cancer centre and to estimate the frequency of utilisation of chemoprophylaxis in these patients. Two retrospective reviews were performed. Over a 10-year period, 14 cases of PCP were identified: no cases were attributable to failed chemoprophylaxis, drug allergy or intolerance. During a 6-month period, 73 patients received high-dose corticosteroid therapy (⩾25 mg prednisolone or ⩾4 mg dexamethasone daily) for ⩾4 weeks. Of these, 22 (30%) had haematologic malignancy, and 51 (70%) had solid tumours. Fewer patients with solid tumours received prophylaxis compared to patients with haematologic malignancy (3.9 *vs* 63.6%, *P*<0.0001). Guidelines for PCP chemoprophylaxis in patients with haematologic malignancy or solid tumours who receive corticosteroid therapy are proposed. Successful primary prevention of PCP in this population will require a multifaceted approach targeting the suboptimal prescribing patterns for chemoprophylaxis.

*Pneumocystis jirovecii* pneumonia (PCP) is a potentially life-threatening opportunistic infection seen predominantly in immunosuppressed individuals, and with increasing frequency in human immunodeficiency (HIV)-negative patients. The case fatality rate among HIV-negative patients with PCP has remained approximately 50% over the last three decades (Walzer *et al*, 1974; Sepkowitz *et al*, 1992; Sepkowitz, 2002), despite identification of risk factors, including malignancy (Sepkowitz *et al*, 1992; Zahar *et al*, 2002), haematologic disorders (Sepkowitz *et al*, 1992), radiation therapy (Mathew and Grossman, 2003), chemotherapy (Hughes *et al*, 1975; Sepkowitz *et al*, 1992), organ transplantation (Hardy *et al*, 1984; Sepkowitz *et al*, 1995) and CD4^+^ lymphopenia (Mansharamani *et al*, 2000). In patients with solid tumours or haematologic malignancy, corticosteroid therapy is the most common predisposing risk factor for developing PCP (Sepkowitz *et al*, 1992; Sepkowitz, 1993; Yale and Limper, 1996).

Identifying patients who are at risk of PCP is important, as highly effective prophylactic strategies are available. Risk assessment based on intensity of corticosteroid therapy is possible as dose, duration and tapering of corticosteroid therapy appear to impact upon the risk for development of PCP (Sepkowitz *et al*, 1992; Slivka *et al*, 1993; Sepkowitz, 1996; Yale and Limper, 1996). In a study of 116 HIV-negative patients with PCP, the median daily dose of corticosteroid was equivalent to 30 mg prednisolone, and the median duration of therapy before the onset of infection was 12 weeks (Yale and Limper, 1996). Sepkowitz *et al* have recommended that any patient with an underlying immunologic disorder (including malignancy) who receives the equivalent of at least 20 mg of prednisolone daily for more than 1 month be given prophylaxis (Sepkowitz *et al*, 1995; Sepkowitz, 1996).

The Peter MacCallum Cancer Centre (PMCC) is a tertiary care cancer centre which sees the full spectrum of malignant disorders. Since 1999, guidelines for trimethoprim–sulphamethoxazole (TMP–SMX) prophylaxis have been utilised in patients receiving selected high-intensity chemotherapy protocols: hyper-CVAD (Kantarjian *et al*, 2000), VAD (Barlogie *et al*, 1984), CEVAD (Giles *et al*, 2000) and FLAG (Estey *et al*, 1994). Adherence to the guidelines and frequency of chemoprophylaxis in other patients with haematological malignancy or solid tumours is unknown.

The aims of this study were to (1) identify the potentially preventable cases of PCP, and (2) estimate the proportional use of suitable chemoprophylaxis in patients at risk for PCP on the basis of corticosteroid therapy. Finally, we propose a strategy for PCP prophylaxis in patients with haematological malignancy or solid tumours, who receive high-dose corticosteroid therapy.

## MATERIALS AND METHODS

To study the relationship between use of prophylaxis and development of PCP, a retrospective case series of all confirmed cases of PCP treated at PMCC between 1994 and 2003 was performed. The proportion of at-risk patients receiving prophylaxis was estimated by a retrospective review of hospital pharmacy records, identifying the use of prophylaxis in patients receiving corticosteroid therapy during a 6-month period (2003).

### *Pneumocystis jirovecii* pneumonia cases

All patients with a discharge diagnosis of PCP from 1/1/1994 to 31/12/2003 were identified by ICD9-CM or ICD10-AM code. A case was defined as a patient with morphologic evidence of *Pneumocystis* by toluidine blue staining and/or direct fluorescent antigen detection in a respiratory specimen (sputum, induced sputum or bronchoscopy specimen). Corticosteroid therapy was defined as administration of prednisolone, dexamethasone, hydrocortisone or methylprednisolone in addition to any chemotherapeutic regimen containing dexamethasone or prednisolone components. Only cases receiving corticosteroid therapy prior to onset of PCP were studied.

Charts were reviewed, and demographic details, clinical findings, predisposing factors and treatment outcome established for each case. Dose of corticosteroid, duration of therapy prior to onset of symptoms and tapering of doses were determined. Other possible risk factors for PCP were analysed: radiation therapy, chemotherapy and stem cell transplantation. Use of prophylaxis (TMP–SMX, dapsone, pentamidine), and potential reasons for lack of prophylaxis (previous drug allergy or intolerance, patient not considered high risk by treating physician, patient requiring palliative care services, noncompliance) were documented.

### Prophylaxis in at-risk patients

Pharmacy records for a 6-month period (1/6/03–30/11/03) were used to identify patients at PMCC who were at risk for development of PCP on the basis of steroid therapy and underlying malignancy. At-risk cases were defined as patients who were dispensed with ⩾25 mg prednisolone or ⩾4 mg dexamethasone daily for ⩾4 weeks. Chart review was performed to determine the demographic details and to identify concurrent use of PCP prophylaxis (as defined above).

### Statistical analysis

Statistical comparison of patients with haematologic malignancy and solid tumours was carried out by the *χ*^2^-test, with *P*<0.05 deemed significant.

## RESULTS

### *Pneumocystis jirovecii* pneumonia cases

Out of 60 patients identified with a discharge diagnosis of PCP, 35 were excluded because no microbiological confirmation of PCP was made. Of the remaining 25 patients with confirmed PCP, 11 had no corticosteroid therapy administered prior to onset of PCP. Two of these had solid tumours (colorectal adenocarcinoma, anaplastic thyroid carcinoma) and nine patients had haematological malignancy (AML (three), non-Hodgkin's lymphoma (two), Hodgkin's disease (two), CLL (two)). In the subgroup with haematological malignancy, a number of predisposing factors were identified: external beam radiation (three), autologous stem cell transplantation (two), and chemotherapy with fludarabine (four), cyclophosphamide (three), cytarabine (three) and methotrexate (one). *P. jirovecii* pneumonia was the cause of death in three out of 11 cases.

Of the 14 patients who received steroid therapy prior to onset of microbiologically confirmed PCP, incomplete data were available for one patient (steroid therapy commenced prior to transfer from another institution). This patient was not included in analysis. Among the 13 analysed cases, the mean age was 56 years (eight males, five females). Table 1 summarises the underlying malignancy and corticosteroid therapy in cases. The median duration of corticosteroid therapy prior to onset of PCP was 35 days (range 18–92), and four cases (31%) were receiving tapering doses at diagnosis of PCP. There were no cases due to failed prophylaxis (no case received prophylaxis prior to onset of PCP), documented drug allergy or intolerance precluding consideration of prophylaxis. One case was receiving palliative care services prior to onset of PCP symptoms. Mortality attributed to PCP was 8%.

### Prophylaxis in at-risk patients

**Figure 1:**
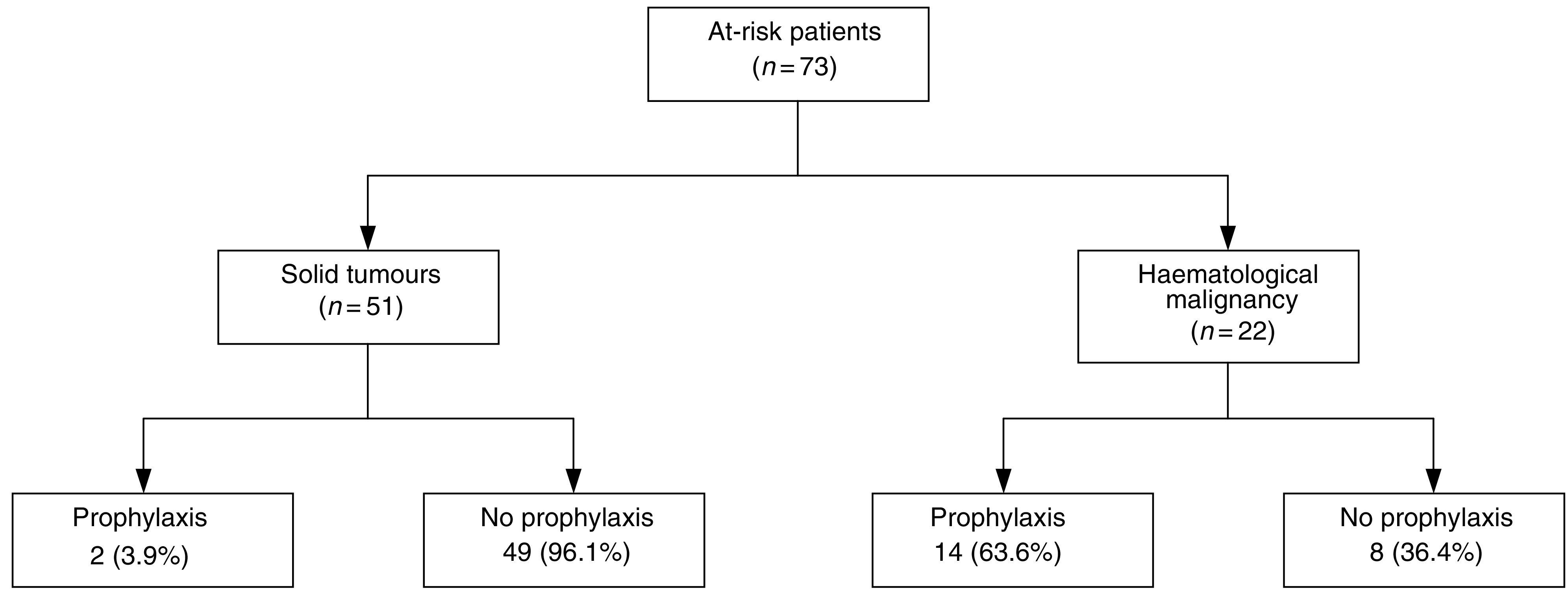
*Pneumocystis jirovecii* pneumonia prophylaxis in at-risk patients receiving corticosteroid therapy, PMCC 1/6/03–30/11/03.

In all, 73 patients receiving corticosteroid therapy of sufficient dose and duration were identified during the defined 6-month period. Of these, 22 (30%) had haematological malignancy and 51 (70%) had solid tumours. Table 2 lists the frequency of diseases in these populations. Figure 1 shows all patients who received ⩾25 mg prednisolone or ⩾4 mg dexamethasone daily for ⩾4 weeks, and the proportion who were also administered PCP prophylaxis. Significantly fewer at-risk patients with solid tumours received prophylaxis compared to patients with haematologic malignancy (3.9 *vs* 63.6%, *P*<0.0001).

During this 6-month period, one case of PCP occurred in a patient not receiving chemoprophylaxis. Using the number of patients receiving corticosteroid therapy without PCP chemoprophylaxis as the denominator (57 patients in 6 months), the estimated crude incidence is 18 PCP cases per 1000 patients with malignancy receiving corticosteroid therapy for ⩾4 weeks.

## DISCUSSION

Our study focused on PCP prophylaxis in patients receiving corticosteroid therapy for solid tumours or haematologic malignancy. Although the mechanisms of immune compromise in this population are multifactorial, corticosteroid therapy is a significant and readily identifiable risk factor, providing opportunity for modification of prescribing practices relevant to prophylaxis.

We have identified differences in proportion of patients with solid tumours receiving chemoprophylaxis compared to patients with haematologic malignancy. This may be attributed to the use of prescriber guidelines within some groups of haematology patients at PMCC. However, even in this group, a large proportion (36.4%) were not prescribed chemoprophylaxis, and the observed number of cases of PCP (seven out of 13) in haematology patients would suggest significant under-prescribing. Our estimation of incidence of PCP is based upon a number of assumptions, including consistency of hospital bed-days, indications for corticosteroid therapy, and proportion of solid and haematological malignancy treated at PMCC during the study period. Community-based prescription of corticosteroid therapy was not captured, leading to possible overestimation of incidence.

In our series, predisposing corticosteroid therapy was not administered in 11 patients with PCP. Only two cases had solid tumours, suggesting that the vast majority of at-risk patients with solid tumours can be identified on the basis of corticosteroid therapy. Nine cases had haematological malignancy. Given the heterogeneity of potential risk factors in this subgroup, distinct recommendations for prophylaxis must be based upon the underlying disease or predisposing chemotherapy. For example, chemoprophylaxis should be administered for 6 months following engraftment in allogeneic transplant recipients (Dykewicz, 2001). This may be extended where immunosuppressive therapy or chronic GVHD persists beyond 6 months. Chemoprophylaxis should be considered in autologous stem cell transplant recipients with underlying lymphoma or leukaemia, intense conditioning regimens, or recently administered fludarabine or 2-chlorodeoxyadenosine (Rodriguez and Fishman, 2004). Patients receiving alemtuzumab therapy should receive PCP chemoprophylaxis for at least 2 months after therapy (Keating *et al*, 2004).

### Trimethoprim–sulfamethoxazole prophylaxis

*Pneumocystis jirovecii* pneumonia prophylaxis is effective in patients not infected with HIV. Munoz *et al* (1997) showed that TMP–SMX (administered on weekends only) eliminated all cases of PCP in a population of heart transplant recipients, compared to 4% incidence prior to prophylaxis. TMP–SMX has been shown to be an effective prophylaxis in patients with cancer (Hughes *et al*, 1977). Daily and thrice weekly dosing regimens have demonstrated equivalence in patients with leukaemia (Hughes *et al*, 1987; Rossi *et al*, 1987), and twice weekly dosing has been shown to be effective in allogeneic transplant recipients (Souza *et al*, 1999).

### Precautions and contraindications

Contraindications to the TMP–SMX prophylaxis must be considered: documented hypersensitivity, megaloblastic anaemia due to folate deficiency, severe renal impairment and porphyria (Masters *et al*, 2003). Caution must also be exercised in patients with impaired renal function, impaired hepatic function, severe drug allergies, glucose-6-phosphate dehydrogenase deficiency or blood dyscrasias. The potential for myelosuppression or drug interactions may necessitate consideration of an alternative prophylactic agent.

Myelosuppression has been associated with TMP–SMX use in children with acute lymphoblastic leukaemia (Woods *et al*, 1984). Conversely, in a study of adult patients with acute leukaemia, no significant difference in myelosuppression was found when TMP–SMX (1 double strength tablet twice daily) was compared with placebo (Ward *et al*, 1993). The original reports of efficacy of TMP–SMX for prophylaxis did not demonstrate significant myelosuppression (Hughes *et al*, 1977, 1987). However, when used for prophylaxis against bacteraemia following autologous bone marrow transplantation, the time to neutrophil recovery is significantly longer in patients receiving TMP–SMX, compared to patients receiving ciprofloxacin (Imrie *et al*, 1995). We recommend that an alternative prophylactic agent be used in at-risk patients with expected myelosuppression >7 days (Table 3).

In patients receiving chemotherapy with methotrexate, pancytopenia may occur if prophylactic TMP–SMX is used concurrently. The sulphamethoxazole component may increase toxicity of methotrexate by displacement from binding sites or reduced renal excretion (Masters *et al*, 2003; Mathew and Grossman, 2003). Small studies of children with acute leukaemia have demonstrated both increased free methotrexate (Ferrazzini *et al*, 1990) and no change in plasma concentration of methotrexate (Beach *et al*, 1981) when co-administered with TMP–SMX. In a study of patients with rheumatoid arthritis treated with up to 25 mg methotrexate per week who received TMP–SMX prophylaxis, no patient developed myelosuppression (Langford *et al*, 2003). Conversely, there are a number of case reports of significant adverse events with the combination (Groenendal and Rampen, 1990; Govert *et al*, 1992; Steuer and Gumpel, 1998; Saravana and Lalukotta, 2003). We therefore recommend an alternative prophylactic agent in the setting of concurrent methotrexate therapy (Table 3).

Prophylaxis with TMP–SMX may not be tolerated in patients with advanced malignancy and reduced oral intake due to nausea or dysphagia. This may not have been a significant contributor to duration of prophylaxis in our series as only one out of 13 cases of PCP were utilising palliative care services at the time of diagnosis. Further study is required to address the question of appropriateness of commencing prophylaxis in the setting of palliation.

### Alternative agents

As demonstrated in HIV-infected patients (Martin *et al*, 1993; Warnock and Rimland, 1996; El-Sadr *et al*, 1998), alternative prophylactic agents may be used, but are associated with higher rates of failure. Dapsone prophylaxis (50 mg three times per week) in allogeneic transplant recipients has a significantly higher incidence of failure when compared to TMP–SMX (Souza *et al*, 1999). Aerosolised pentamidine (150 mg every 2 weeks or 300 mg per month) used after bone marrow transplantation has been shown to be inferior to TMP–SMX for PCP prophylaxis (Vasconcelles *et al*, 2000) and associated with an increased risk for developing toxoplasmosis in immunocompromised patients with antibodies to *Toxoplasma gondii* (Machado *et al*, 1998). Safety and tolerability of atovaquone prophylaxis (1500 mg daily) has been demonstrated in autologous stem cell transplant recipients (Colby *et al*, 1999), but studies of efficacy in non-HIV-infected patients are lacking. Use of clindamycin (300 mg daily) and primaquine (15 mg daily) for prophylaxis in HIV-infected patients is associated with a higher risk for developing PCP than the TMP–SMX prophylaxis (Barber *et al*, 1996). Efficacy of clindamycin/primaquine for prophylaxis in patients with malignancy has not been reported.

Due to ease of administration and medical costs, we recommend that dapsone be used as second-line prophylaxis if TMP–SMX is contraindicated or not tolerated (Table 3). Nebulised pentamidine may also be used, and atovaquone may be considered in patients unable to tolerate TMP–SMX or dapsone.

### Duration of prophylaxis

Although some have recommended that prophylaxis continue for 1 month after discontinuation of corticosteroids (Sepkowitz, 2002), extended duration may be required with other concurrently administered immunosuppressive therapies. For example, cytarabine (Hughes *et al*, 1975), cyclophosphamide (Kulke and Vance, 1997), methotrexate (Kane *et al*, 1993), fluorouracil (Hardy *et al*, 1987) and fludarabine (Bastion *et al*, 1991) have all been associated with development of PCP in the absence of corticosteroid treatment, although the absolute risk is unclear. We recommend continued primary prophylaxis or lifelong secondary prophylaxis in the setting of ongoing immunosuppression (Table 3).

### Improving utilisation and prescriber practices

Suboptimal prescribing patterns for PCP chemoprophylaxis have been described in HIV-infected patients (Montaner *et al*, 1996; Schwarcz *et al*, 1997). Reasons are multifactorial, including patient reluctance or inability to access healthcare services (Schwarcz *et al*, 1997), and failure of a treating clinician to identify the need for prophylaxis (Auperin *et al*, 1994). In our patient population, it appears that prescriber factors are a greater barrier to PCP prophylaxis than poor access to healthcare, as no case of noncompliance was identified in the series of PCP cases, and patients were otherwise attending the regular review for treatment of underlying malignancy.

We have formulated guidelines for PCP chemoprophylaxis in patients receiving corticosteroid therapy for malignancy (Table 3). If applied to the cases of PCP observed at PMCC, 11 out of 13 cases (85%) could potentially have been prevented. Successful primary prevention of PCP in this population will require a multifaceted approach including academic detailing (one-on-one education of clinicians and hospital pharmacists). This has previously been successfully implemented to enhance the use of preventive medication for corticosteroid-induced osteoporosis (Naunton *et al*, 2004). Novel strategies, such as generation of computerised clinical reminders (Kralj *et al*, 2003) from pharmacy records of dispensed corticosteroid, should also be considered.

## Figures and Tables

**Table 1:** Cases of PCP in patients receiving corticosteroid therapy, PMCC 1/1/1994–31/12/2003

		**Corticosteroid^a^**			**Other risk factors for PCP**			
**Age/sex**	**Malignancy**	**Dose**	**No. of days**	**Tapering**	**Transplantation**	**Cytotoxic therapy**	**External beam radiation**	**Outcome**
*Solid tumours*								
57/M	Thyroid carcinoma	DXM 16 mg	30	Y	N	Radioactive iodine	N	Resolved
63/M	GBM	DXM 16 mg	34	N	N	N	N	Resolved
76/M	NSCLC	DXM 12 mg	36	N	N	Gemcitabine carboplatin	N	Resolved
64/M	SCLC	DXM 16 mg	28	N	N	Carboplatin, paclitaxel	N	Died
36/F	Breast carcinoma	PNL 25 mg	40	N	Autologous HSCT	EC	Y	Resolved
65/M	NSCLC	DXM 16 mg	88	Y	N	Gemcitabine	Y	Resolved
								
*Haematologic malignancy*								
44/F	NHL	PNL 100 mg	50	N	N	CHOP	Y	Resolved
71/M	AML	PNL 25 mg	45	N	N	FLAG, hydroxyurea	Y	Resolved
61/F	NHL	DXM 16 mg	35	Y	N	CHOP, Mabthera, RICE	N	Resolved
68/F	NHL (cerebral metastases)	DXM 8 mg	92	N	N	CHOP, Mabthera, IT MTX, Ara-C	N	Resolved
18/M	Burkitt's lymphoma	DXM 40 mg	18	N	N	MTX,Ara-C	Y	Resolved
48/F	Multiple myeloma	DXM 100 mg	—b	Y	N	VAD, cyclophosphamide	N	Resolved
41/M	Multiple myeloma	DXM 16 mg	25	Y	Autologous HSCT	PCAB, VAD, melphalan	N	Resolved
64/F	CML	PNL 25 mg	33	N	N	IFN, hydroxyurea, busulphan, Ara-C, idarubicin, fludarabine	N	Resolved

a Initial daily dose and number of days of corticosteroid therapy prior to onset of PCP symptoms; DXM=dexamethasone; PNL=prednisolone.

b Patient commenced corticosteroid therapy at another institution prior to transfer.

GBM=glioblastoma multiforme; NHL=non-Hodgkin's lymphoma; AML=acute myeloid leukaemia; CML=chronic myeloid leukaemia; EC=epirubicin, cyclophosphamide; CHOP=cyclophosphamide, doxorubicin, oncovin, prednisolone; FLAG=fludarabine, cytarabine, G-CSF; RICE=rituximab, ifosfamide, carboplatin, etoposide; MTX=methotrexate; Ara-C=cytarabine; IFN=interferon *α*-2b; PCAB=prednisolone, cyclophosphamide, doxorubicin, carmustine; VAD=vincristine, doxorubicin, dexamethasone; HSCT=haematopoietic stem cell transplantation.

**Table 2:** At-risk patients receiving high-dose corticosteroid therapy, PMCC 1/6/03–30/11/03

**Malignancy**	***n***
*Solid tumours*	
Lung (non-small cell)	13
Genitourinary	9
Breast	8
Gastrointestinal	7
Melanoma	4
Adenocarcinoma, unknown primary	4
Central nervous system (primary)	2
Sarcoma	2
Adenoid cystic carcinoma	1
Parotid	1
	
*Haematologic malignancy*	
Multiple myeloma	14
Non-Hodgkin's lymphoma	6
Chronic lymphocytic leukaemia	2
	
Total	73

**Table 3:** Guidelines: PCP prophylaxis for patients with malignancy who receive corticosteroid therapy

Chemoprophylaxis for *Pneumocystis jirovecii* pneumonia should be administered when treatment with ⩾20 mg prednisolone equivalents for ⩾1 month is planned.	
First-line prophylactic agent should be trimethoprim–sulphamethoxazole (one DS^a^ tablet daily, one DS tablet three times weekly or two DS tablets twice weekly), unless:	
(i)	Myelosuppression due to chemotherapy or radiation likely to be present for >7 days
	Recommend: second-line prophylactic agent
(ii)	Previous allergy or hypersensitivity to sulpha-drugs
	Recommend: trimethoprim–sulphamethoxazole desensitisation (unless previous anaphylaxis)
(iii)	Planned methotrexate chemotherapy
	Recommend: second-line prophylactic agent.
	
A second-line prophylactic agent should be used if trimethoprim–sulphamethoxazole is contraindicated:	
(i)	Dapsone (100 mg daily), OR
(ii)	Pentamidine^b^ (nebulised, 300 mg monthly), OR
(iii)	Atovaquone^c^ (1500 mg daily).
Prophylaxis should continue until at least 1 month after steroid cessation. A longer period of prophylaxis may be required if ongoing chemotherapy (e.g. cytarabine, cyclophosphamide, fludarabine, fluorouracil, methotrexate) is planned. Life-long prophylaxis should be considered if the patient has had a previous episode of PCP and persisting immunosuppression.	

a DS=double strength; trimethoprim 160 mg per 800 mg sulphamethoxazole.

b Screening antibodies for *T. gondii* should be checked prior to use following bone marrow transplantation.

c No studies of efficacy in patients with malignancy.
